# Research of Fluorescent Properties of a New Type of Phosphor with Mn^2+^-Doped Ca_2_SiO_4_

**DOI:** 10.3390/s21082788

**Published:** 2021-04-15

**Authors:** Xiaozhou Fan, Wenqi Zhang, Fangcheng Lü, Yueyi Sui, Jiaxue Wang, Ziqiang Xu

**Affiliations:** 1 Department of Electrical Engineering, College of Electrical and Electronic Engineering, North China Electric Power University, Baoding 071003, China; 18331130993@163.com (X.F.); lfc_ncepu1958@163.com (F.L.); suiyueyi1996@163.com (Y.S.); wjx@ncepu.edu.cn (J.W.); 2 Hebei Provincial Key Laboratory of Power Transmission Equipment Security Defense, North China Electric Power University, Baoding 071003, China; 3 State Grid Nanjing Power Supply Company, Nanjing 210000, China; sgcc_xzq@163.com

**Keywords:** fluorescent optical fiber, fluorescence intensity, lifetime, temperature sensor

## Abstract

Fluorescent optical fiber temperature sensors have attracted extensive attention due to their strong anti-electromagnetic interference ability, good high-voltage insulation performance, and fast response speed. The fluorescent material of the sensor probe directly determines the temperature measurement effect. In this paper, a new type of fluorescent material with a Mn^2+^-doped Ca_2_SiO_4_ phosphor (CSO:Mn^2+^) is synthesized via the solid-state reaction method at 1450 °C. The X-ray diffraction spectrum shows that the sintered sample has a pure phase structure, although the diffraction peaks show a slight shift when dopants are added. The temperature dependence of the fluorescence intensity and lifetime in the range from 290 to 450 K is explored with the help of a fluorescence spectrometer. Green emission bands peaking at 475 and 550 nm from Mn^2+^ are observed in the fluorescence spectra, and the intensity of emitted light decreases as the temperature rises. The average lifetime of CSO:Mn^2+^ is 17 ms, which is much higher than the commonly used fluorescent materials on the market. The fluorescence lifetime decreases with increasing temperature and shows a good linear relationship within a certain temperature range. The research results are of great significance to the development of a new generation of fluorescence sensors.

## 1. Introduction

As one of the fundamental physical parameters, temperature is of great significance in scientific research, industrial and agricultural production, national defense modernization, and other fields [1]. According to different temperature measurement principles, there are three main types of thermometers: liquid thermometers based on thermal expansion of materials [2], thermocouple thermometers based on the Seebeck effect [3], and optical thermometers, including fluorescent temperature measurement systems [4]. With the continuous development of industry and science, many temperature measurement systems face increasingly harsh environments, and higher requirements are placed on these systems. In recent years, optical fiber temperature sensors have received widespread attention due to their strong anti-electromagnetic interference ability, high-voltage insulation, fast response speed, and non-contact measurement [5]. According to different optical principles, optical fiber temperature sensors can be divided into the following types: (1) based on fluorescence signals and fluorescence temperature-measuring devices with temperature information correlation [6]; (2) based on optical interference principles such as F-P interference, thin-film interference, and white light interference [7,8]; (3) based on the light absorption characteristics of semiconductors such as gallium arsenide [9]; (4) based on the thermal light radiation of black-body cavity, quartz, and light guide rods [10]; and (5) based on the light-carrying temperature information forming Raman scattering and Rayleigh scattering in the light [11]. Among them, fluorescent optical fibers have become a research hotspot in the field of optical fiber temperature measurement. Generally, all the temperature-dependent fluorescent properties, such as excitation spectra, emission spectra, and decay lifetime, can be exploited to detect temperature due to their variations with temperature [12,13,14,15]. The generated fluorescence can penetrate media so that real-time and remote operations can be realized, which makes the luminescence-based measurement more competitive. According to the different temperature measurement principles, sensors are mainly divided into fluorescence-intensity-type, fluorescence-proportional-type, and fluorescence lifetime sensors [16]. Compared with the first two types, the fluorescence lifetime sensor is not affected by the fluctuation of the excitation light source, and the temperature measurement is realized only according to the length of the fluorescence afterglow lifetime [17].

The duration of the fluorescence afterglow depends on the optical temperature characteristics of the fluorescent substance located on the temperature probe [18]. At present, most of the temperature measurement research is concentrated on the fluorescent material doped with rare earth ions, Dy^3+^, Tm^3+^, Nd^3+^, and Eu^3+^, and other rare earth ions have been successfully applied in fluorescent probe materials [19,20,21,22,23]. However, rare earth elements are expensive due to their small abundance, and not all of their fluorescence characteristics are feasible or sufficiently sensitive. In recent years, researchers have discovered that some transition metals can also become luminescence-activating ions or sensitizing ions, which exchange energy with the luminescence center in the matrix to increase luminescence intensity [24,25,26]. For example, Wu [27] prepared Cr^3+^-doped spinel fluorescent materials and explored the relationship between the fluorescence lifetime and temperature. At room temperature, the fluorescence lifetime of the most commonly used ruby is 4.2 ms, that of YAG:Cr^3+^ is 2 ms, and that of MgAl_2_O_4_:Cr^3+^ is 10 ms. Chi [28] developed a new type of fluorescent material with Mn^2+^-doped ZnGeO_4_ and tested the fluorescence intensity and lifetime in the temperature range of 250 to 420 K; a maximum relative sensitivity of 12.2% K^−1^ was achieved, and the best temperature resolution was about 0.68 K. Wang et al. [29] added Zn ions to ZnO, which improved the ability of the trap to capture electrons and enhanced the luminescence intensity of the material. Researchers added Fe^3+^ ions to LiAlO_2_ or Co and Ni ions to ZnGa_2_O_4_ to generate near-infrared light [30,31]. The transition metal Mn has rich chemical valence states and can be used alone in either organic luminescent materials or inorganic luminescent materials [32]. In addition, the d-d orbital transition of Mn^2+^ belongs is a spin-forbidden transition, so when Mn^2+^ is used as an active ion, its fluorescence decays slowly and the fluorescence lifetime is usually on the order of milliseconds, which is convenient for measurement [33].

In the choice of a phosphor matrix, silicate has excellent physical, chemical, and thermal stability; moreover, the cost is low, which is suitable for preparing phosphors with excellent performance, so rare-earth-doped silicate phosphors are subject to extensive research [34]. Sato et al. [35] developed a Ca_2_SiO_4_:Eu^2+^ phosphor in 2014, whose fluorescence lifetime was 2.5 ms. This phosphor realizes a broad spectrum of yellow light emission under 365 nm excitation, and with an increase of Eu^2+^ doping, the emission peak of the spectrum gradually shifts to red. Kalaji et al. [36] reported a Ca_2_SiO_4_:Ce^3+^ phosphor, and this phosphor is excited at 450 nm and has an emission peak at 565 nm, which is a new type of yellow phosphor. In this paper, Ca_2_SiO_4_ (CSO) is used as the phosphor matrix, and a new type of fluorescent material (CSO:Mn^2+^) is synthesized by doping with the transition metal Mn^2+^. The dependence between the optical properties of the material and the temperature change is explored, and a good linear relationship between the fluorescence lifetime and temperature change of the material is found. The findings are of great significance to the development of a new generation of fluorescent optical fiber temperature sensors.

## 2. Materials and Methods

### 2.1. Synthesis

Pure and 2% Mn^2+^-doped CSO phosphors were synthesized via the high-temperature solid-phase method. High-purity SiO_2_ (AR), CaO (AR), and MnCO_3_ (AR) raw materials were accurately weighed according to a certain stoichiometric ratio. Stoichiometric mixtures of raw powders were placed in a corundum mortar and fully ground for 2 h, and then the obtained powder was put into a mold and pressed by a tablet press with 20 MPa pressure for 1 min to form a disc with a diameter of 15 mm. Subsequently, the obtained sample was sintered in air at 1450 °C for 4 h, the final product was cooled to room temperature, and a fluorescent material sample was obtained.

### 2.2. Experimental Setup

The crystal structure of the sample was tested using a Rigaku UItimate IV X-ray diffractometer (Cu/Kα radiation) with a scanning step of 0.02° in the 2θ range from 20° to 70°. The excitation spectrum, emission spectrum, and fluorescence lifetime of the sample were tested using an Edinburgh FLS1000 fluorescence spectrometer with a 450 W ozone-free xenon arc lamp that covers a range of 230 to 1000 nm for steady-state measurements. The time resolution of the fluorescence spectrometer is 1e-9s (nanosecond level), which can meet the measurement range of fluorescence attenuation. To explore the dependence between the optical properties and temperature changes, temperature control was realized using a temperature controller (OMRON E5CC-800) with a type-K thermocouple and a heating tube.

## 3. Results and Discussion

### 3.1. Structure Properties

**Figure 1:**
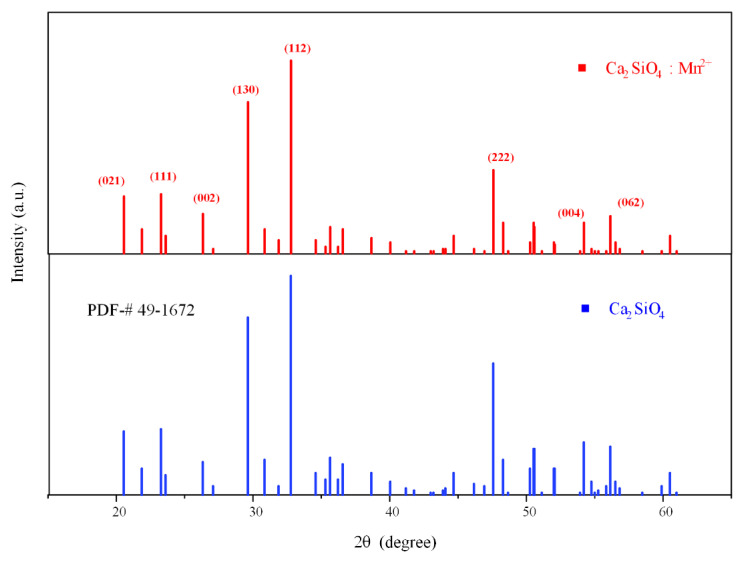
The XRD pattern of Ca_2_SiO_4_ (CSO):Mn^2+^ and the standard PDF of CSO.

The synthesized fluorescent material was characterized with an X-ray diffraction (XRD) diffractometer, and the obtained diffraction peaks were compared with the standard Powder Diffraction File (PDF) #49-1672 of Ca_2_SiO_4_. As shown in Figure 1, the diffraction peaks of the material, especially the three main peaks distributed at 29.7°, 33.2°, and 47.4°, were well matched, which proves that Ca_2_SiO_4_ is generated when the reactants react fully in the high-temperature solid-phase process, and doping with Mn^2+^ does not change the matrix phase of Ca_2_SiO_4_, indicating that all the samples synthesized at 1450 °C are in the pure phase. A small part of the weak impurity peaks in the picture may be derived from impurities such as calcium oxide with insufficient reaction. The crystallographic planes corresponding to the main diffraction peaks are shown in the figure, and the crystal planes corresponding to the three main peaks are (112), (130), and (222). By comparing the existing unit cell parameters in the crystallography open database (COD), we found that the crystal lattice parameters of the sample and γ-Ca2SiO4 (COD: 1546025) are consistent; hence, the crystal form of Ca_2_SiO_4_ synthesized is γ-type.

**Figure 2:**
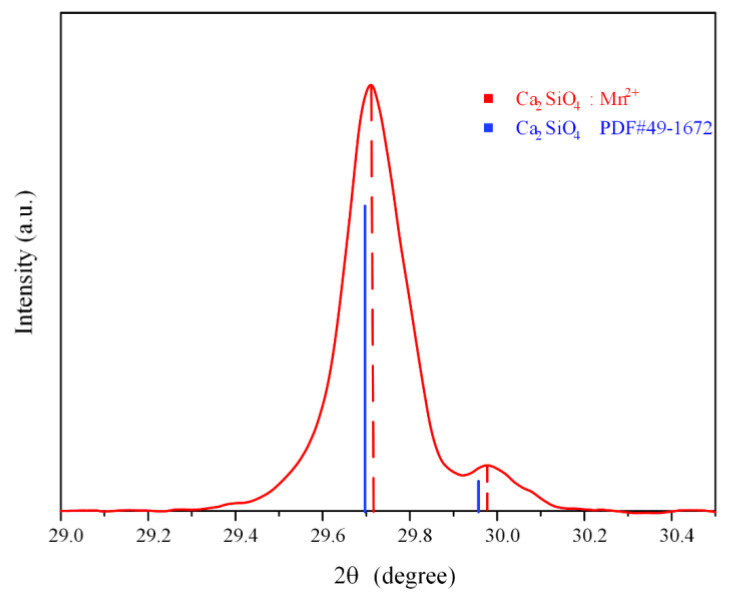
Peak shift of CSO:Mn^2+^ in the range of 29° to 30°.

As shown in Figure 2, it is worth noting that the main peak of CSO:Mn^2+^ distributed at 29.7° is slightly shifted back relative to the standard peak position, and the shift of the diffraction peak implies that the crystal lattice of the doped sample has been shrunk. According to the Bragg equation, 2dsin θ = nλ, where d is the interplanar spacing, n is the reflection order, and λ is the incident wavelength. Since the radius of the manganese ion (0.66Å) is smaller than the radius of the calcium ion (0.1Å), when Mn^2+^ replaces Ca^2+^ sites in the dicalcium silicate lattice, the lattice shrinks and the interplanar spacing d decreases, so the diffraction angle θ increases. The unit cell parameters of pure and Mn^2+^-doped CSO samples were calculated, and the results are given in Table 1.

### 3.2. Fluorescence Spectrum and Lifetime

**Figure 3:**
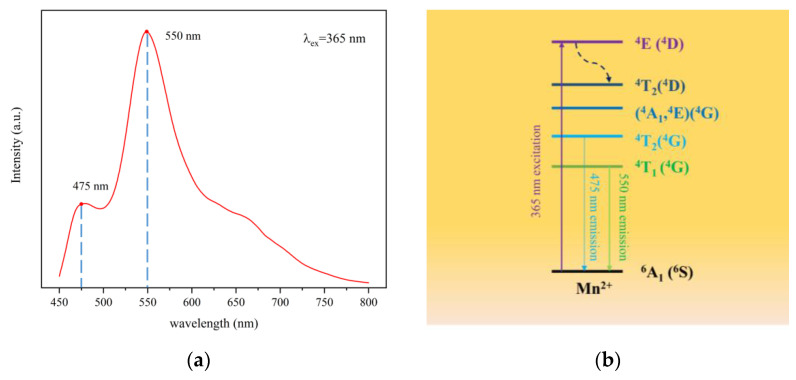
(**a**) The excitation spectrum of CSO:Mn^2+^ under light excitation at 365 nm. (**b**) Energy-level transition diagram of CSO:Mn^2+^.

Under 365 nm excitation, the excitation spectrum of CSO:Mn^2+^ is as shown in Figure 3a. The material has two obvious excitation peaks at 475 and 550 nm, and the peak intensity at 475 nm is obviously smaller than that at 550 nm.

Figure 3b describes the energy-level state diagram of Mn^2+^-doped Ca_2_SiO_4_ [37]. Under laser excitation at 365 nm, the extranuclear electrons transition from the ground state ^6^A_1_ (^6^S) to the excited state ^4^E (^4^D), and then the electrons fall back from the lowest excited state ^4^T_1_ (^4^G) to the ground state ^6^A_1_(^6^S) due to the band-gap transition between the d-d energy levels and emit 550 nm yellow-green light. A small part of the electrons transition from the ^4^T_2_ (^4^G) energy level to the ground state ^6^A_1_ (^6^S) to emit blue-green light. The number of electrons limited by the transition is small, so the light intensity is relatively weak.

**Figure 4:**
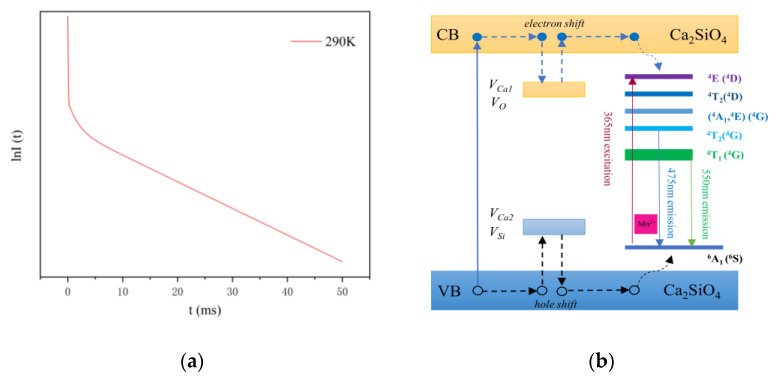
(**a**) The fluorescence intensity decay curve of CSO:Mn^2+^. (**b**) Energy-level diagram and the long persistent fluorescence mechanism of CSO:Mn^2+^.

As shown in Figure 4a, when the excitation light source stops exciting, the fluorescence intensity of CSO:Mn^2+^ drops suddenly. However, the fluorescence decay time remains long, the decay curve can be well fitted via an exponential Equation (1), and the average fluorescence lifetime of the material is calculated by Equation (2).

(1) $$I(t)=A_{1}\exp(-\frac{t_{1}}{\tau_{1}})+A_{2}\exp(-\frac{t_{2}}{\tau_{2}})+A_{3}\exp(-\frac{t_{3}}{\tau_{3}})$$

(2) $$\tau_{a}=\frac{A_{1}\cdot \tau_{1}^{2}+A_{2}\cdot \tau_{2}^{2}+A_{3}\cdot \tau_{3}^{2}}{A_{1}\cdot \tau_{1}^{}+A_{2}\cdot \tau_{2}^{}+A_{3}\cdot \tau_{3}^{}}$$

where I is the fluorescence intensity; A_1_, A_2_, and A_3_ are constants; t is time; τ_1_, τ_2_, and τ_3_ represent the fluorescence lifetimes; and τ_a_ represents the average fluorescence lifetime. After calculation, the average fluorescence lifetime of CSO:Mn^2+^ at 290 K is 17 ms, which is far beyond the fluorescence lifetime of similar materials [27,35].

At 290 K, the values of τ_1_*,* τ_2_, and τ_3_ are 0.047, 1.495, and 18.48 ms, respectively. A_1_, A_2_, and A_3_ are 691.2637, 62.5746, and 85.9381, respectively, which represent the signal strength of each part, and it can be seen that the signal strength of τ_1_ is much greater than that of τ_2_ and τ_3_, which is the main process of transition. According to the transition principle, the τ_1_ process corresponds to the radiation transition, and the electrons return to the ground state directly from the excited state; hence the fluorescence lifetime is shorter. The processes of τ_2_ and τ_3_ correspond to the trap transitions, and when electrons in the excited state are captured by deep and shallow traps on the crystal surface, instant transitions often cannot occur. After a period of time, the electrons in the shallow traps are released by thermal energy or other disturbances, returning to the ground state and producing fluorescence, while electrons in deep traps often take longer.

To show the fluorescence decay process more intuitively, the lnI(t) function was used to express the relationship between the fluorescence intensity I and the time t, as shown in Figure 4a. It can be roughly seen that the curve contains three linear relationships, which correspond to the third-order fitting process in Equation (1).

As shown in Figure 4b, under light excitation at 365 nm, most excitation energy is transferred to the fluorescent centers, followed by the transition of Mn^2+^: ^4^T_1_(^4^G) to the ground state ^6^A_1_(^6^S). When electrons in the conduction band (CB) are captured by electron traps caused by interstitial Ca defects (V_Ca1_) and oxygen vacancies (V_O_), which are intrinsic defects of Ca_2_SiO_4_, and holes in the valence band (VB) are captured by cation vacancies V_Ca2_ and traps V_Si_, part of the energy is stored. Since the traps for storing energy in the lattice are far apart, the electrons and holes do not recombine in a short time. However, under the disturbance of thermal energy, the electrons escape from traps and transfer via the CB to the fluorescent centers or directly tunnel in an inefficient and slow way to the excited states of Mn^2+^. At the same time, the holes move from the traps to the ground state of Mn^2+^ via the VB. The gradual recombination of electrons and holes produces the long persistent fluorescence.

### 3.3. The Relationship between Spectrum and Lifetime with Temperature

**Figure 5:**
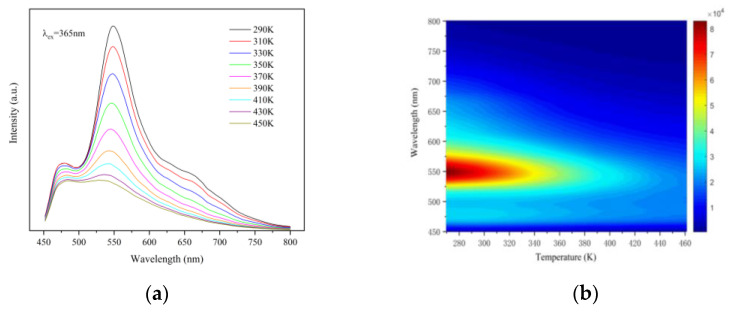
(**a**) The temperature-dependent emission spectra of CSO:Mn^2+^ under laser excitation at 365 nm, with the temperature increasing from 290 to 450 K. (**b**) The corresponding temperature-dependent mapping of CSO:Mn^2+^ under laser excitation at 365 nm.

To further explore the dependence of the optical properties of synthetic materials on temperature, we tested the excitation spectrum and fluorescence lifetime of materials in the range of 290 to 450 K. Figure 5a shows the temperature-dependent emission spectra of CSO:Mn^2+^ under laser excitation at 365 nm with the temperature increasing from 290 to 450 K. There are two broad emission bands ranging from 450 to 600 nm, with a maximum centered at 475 and 550 nm, and the intensity of the two peaks decreases significantly with increasing temperature. The peak intensity of the emission spectrum is highest at 290 K and lowest at 450 K, only 1/4 of the maximum. Figure 5b shows the corresponding temperature-dependent mapping of CSO:Mn^2+^ under laser excitation at 365 nm.

**Figure 6:**
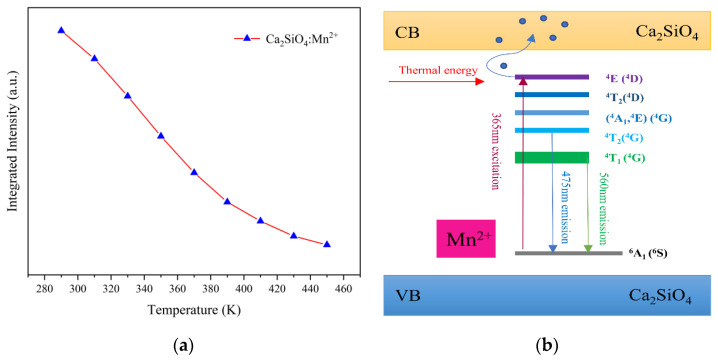
(**a**) Temperature dependence of the integrated intensity of CSO:Mn^2+^. (**b**) Energy-level diagram and the fluorescence process of CSO:Mn^2+^.

As shown in Figure 6a, the integrated fluorescence intensity of the material decays with increasing temperature, indicating that CSO:Mn^2+^ has inferior thermal stability. The phenomenon can be explained by thermal quenching theory. As shown in Figure 6b, under light excitation at 365 nm, electrons transition from the ground ^6^A_1_ (^6^S) state to the excited state ^4^E (^4^D). When the temperature rises, the excited-state electrons absorb thermal energy and jump to the conduction band, instead of light emission. The free electrons in the conduction band can be captured by traps on the surface of the material, and only a small part of the electrons can move back to the ground state, so the intensity of the emitted light is greatly reduced, and even fluorescence quenching occurs.

Relative sensitivity S_R_ is often used to measure temperature and measurement accuracy. It can be defined by the relationship between emission intensity and temperature as Equation (3).

(3) $$S_{RI}=\frac{1}{I}\left|\frac{dI}{dT}\right|\times 100\%$$

(4) $$S_{R\tau }=\frac{1}{\tau }\left|\frac{d\tau }{dT}\right|\times 100\%$$

**Figure 7:**
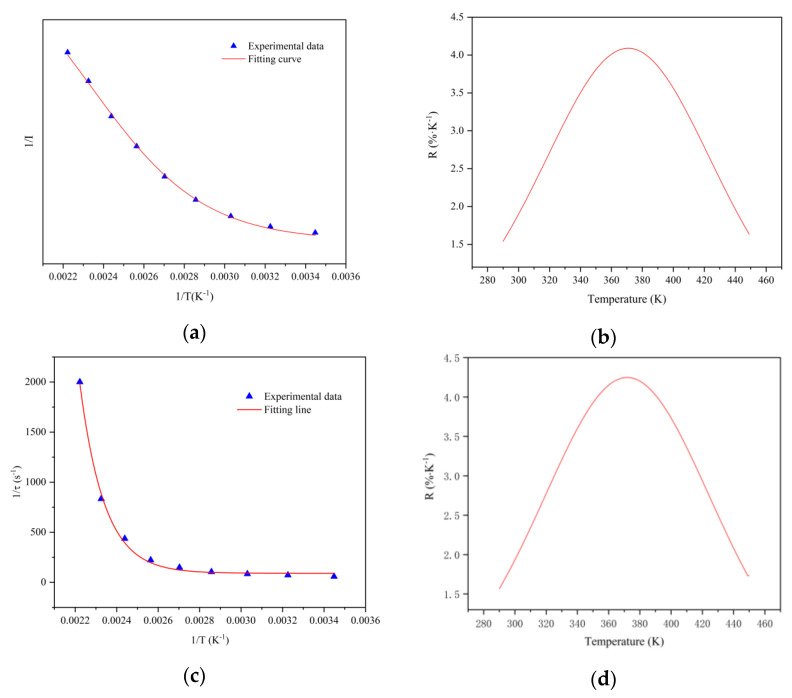
(**a**) The relationship between 1/I and 1/T of CSO:Mn^2+^ at 290–450 K. (**b**) The relationship between relative sensitivity of fluorescence intensity and temperature. (**c**) The relationship between 1/τ and 1/T of CSO:Mn^2+^ at 290–450 K. (**d**) The relationship between relative sensitivity of fluorescence lifetime and temperature.

As shown in Figure 7a, the relationship between 1/I (I represents the integrated emission intensity from 475 to 800 nm) and 1/T (T is the temperature from 290 to 450 K) is gained, which can be fitted with the Arrhenius-type equation. According to Equation (3), the relative sensitivity S_RI_ of the fluorescence intensity with temperature can be calculated, and the variation of relative sensitivity with temperature is shown in Figure 7b. The relationship between 1/τ (τ represents the fluorescence lifetime) and 1/T (T is the temperature from 290 to 450 K) is shown in Figure 7c, the relative sensitivity S_R__τ_ is calculated according to Equation (4), and the variation is shown in Figure 7d. It can be seen that at the temperature range of 330 to 410 K, all relative sensitivities are higher than 3%, the maximum S_RI_ reaches 4.18% at 370 K, and the maximum S_R__τ_ reaches 4.25% at 375 K, which are much better than previously reported rare-earth-ion-doped temperature sensors [38,39,40,41].

**Figure 8:**
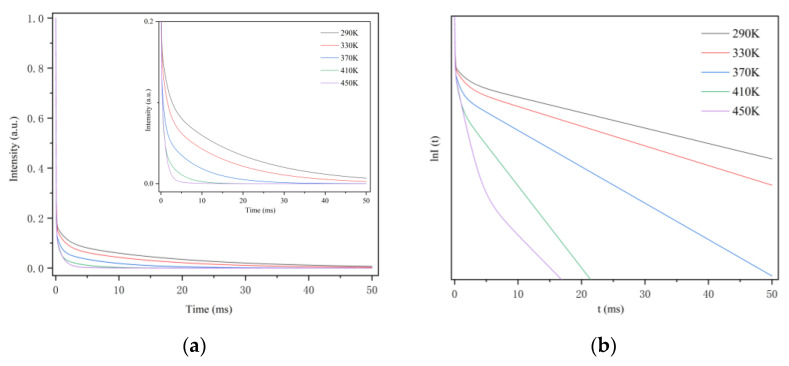
(**a**) The fluorescence afterglow decay curve of CSO:Mn^2+^ at different temperatures. (**b**) The relationship between the semi-logarithmic of I and t at different temperatures.

The fluorescence lifetime of CSO:Mn^2+^ from 290 to 450 K was further measured. Figure 8a shows that when the excitation light stops, the decay curve consists of rapid attenuation and a long persistent decay process. To more intuitively show the relationship between the fluorescence lifetime and the temperature, the coordinate of the diagram was represented by the semi-logarithmic lnI(t), as shown in Figure 8b. It can be seen clearly that as the temperature rises, the decay rate of the curve continues to increase. The mechanism of fluorescence afterglow decay has been explained above: as the temperature increases, the recombination of electrons and holes intensifies under the disturbance of thermal energy, which promotes the increase of the fluorescence afterglow decay rate. 

**Figure 9:**
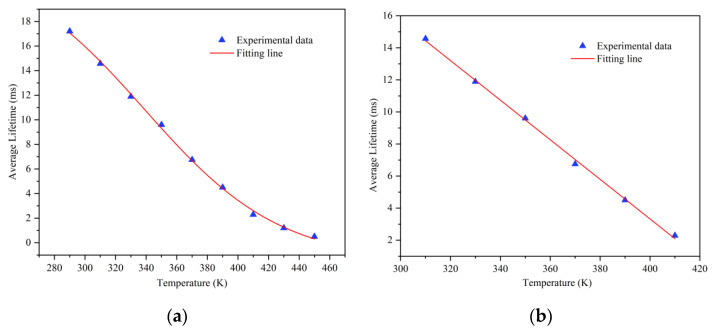
(**a**) The relationship between fluorescence lifetime and temperature in the range of 290 to 450 K. (**b**) The relationship between fluorescence lifetime and temperature in the range of 310 to 410 K.

The average fluorescence lifetime of the material at different temperatures was calculated and can be fitted well by the Boltzmann function, as Figure 9 shows. At the temperature range of 310 to 410 K, the curve of the fluorescence lifetime and temperature is almost linear (R = 0.9892), which provides a good foundation for subsequent fluorescence temperature measurement work.

## 4. Conclusions

In this paper, 2% Mn^2+^-doped Ca_2_SiO_4_ was successfully synthesized via the high-temperature solid-state method. X-ray powder diffraction was performed to prove the synthetic sample has a pure phase. The excitation spectrum, emission spectrum, and fluorescence lifetime of the sample were tested to explore its fluorescent properties. Under light excitation at 365 nm, the sample has two emission peaks at 475 and 550 nm, corresponding to the parity-forbidden d-d band transition of Mn^2+^: ^4^T_2_ to ^6^A_1_ and ^4^T_1_ to ^6^A_1_. The average fluorescence lifetime of the sample at room temperature is 17 ms, which far exceeds the commonly fluorescent materials. To further observe the relationship between the fluorescence characteristics and the temperature, the fluorescence intensity and fluorescence lifetime of the sample in the temperature range of 290 to 450 K were tested. As the temperature increases, the fluorescence intensity of the sample continues to decrease, and the maximum relative sensitivity reaches 4.18% K^−1^ at 370 K. This phenomenon can be explained as electrons in the excited state absorbing thermal energy and jumping to the conduction band instead of the emission band. The fluorescence lifetime of the sample decreases as the temperature rises, and the maximum relative sensitivity reaches 4.25% K^−1^ at 375 K, which is caused by the increase in the recombination rate of electrons and holes in the defect levels (V_Ca1_*,* V_O_ and V_Ca2_*,* V_Si_) under the disturbance of thermal energy. It is worth mentioning that the curve of the fluorescence lifetime at the temperature of 290–450 K can be fitted well with the Boltzmann function, and in the temperature range of 310–410 K, the change trend shows an excellent linear relationship, which may have some value for the development of new fluorescent temperature measurement materials.

## Figures and Tables

**Table 1:** The calculated lattice parameters of pure and 2% Mn^2+^-doped Ca_2_SiO_4_ samples.

Sample	a (Å)	b (Å)	c (Å)	V (Å^3^)
CSO	5.076	11.214	6.758	384.7
CSO:Mn^2+^	5.032	11.173	6.701	376.4
